# Supplementary material for: Pediatric COVID-19 patients in South Brazil show abundant viral mRNA and strong specific anti-viral responses
Source: Nat Commun. 2021 Nov 25;12:6844. doi: 10.1038/s41467-021-27120-y (PMC8617275; doi:10.1038/s41467-021-27120-y)
Supplement: Supplementary file 1 — Supplementary Information [file 41467_2021_27120_MOESM1_ESM.pdf]

Pediatric COVID-19 patients in South Brazil show abundant viral mRNA and strong specific anti-viral responses

Fazolo, T and Bonorino, C et al.

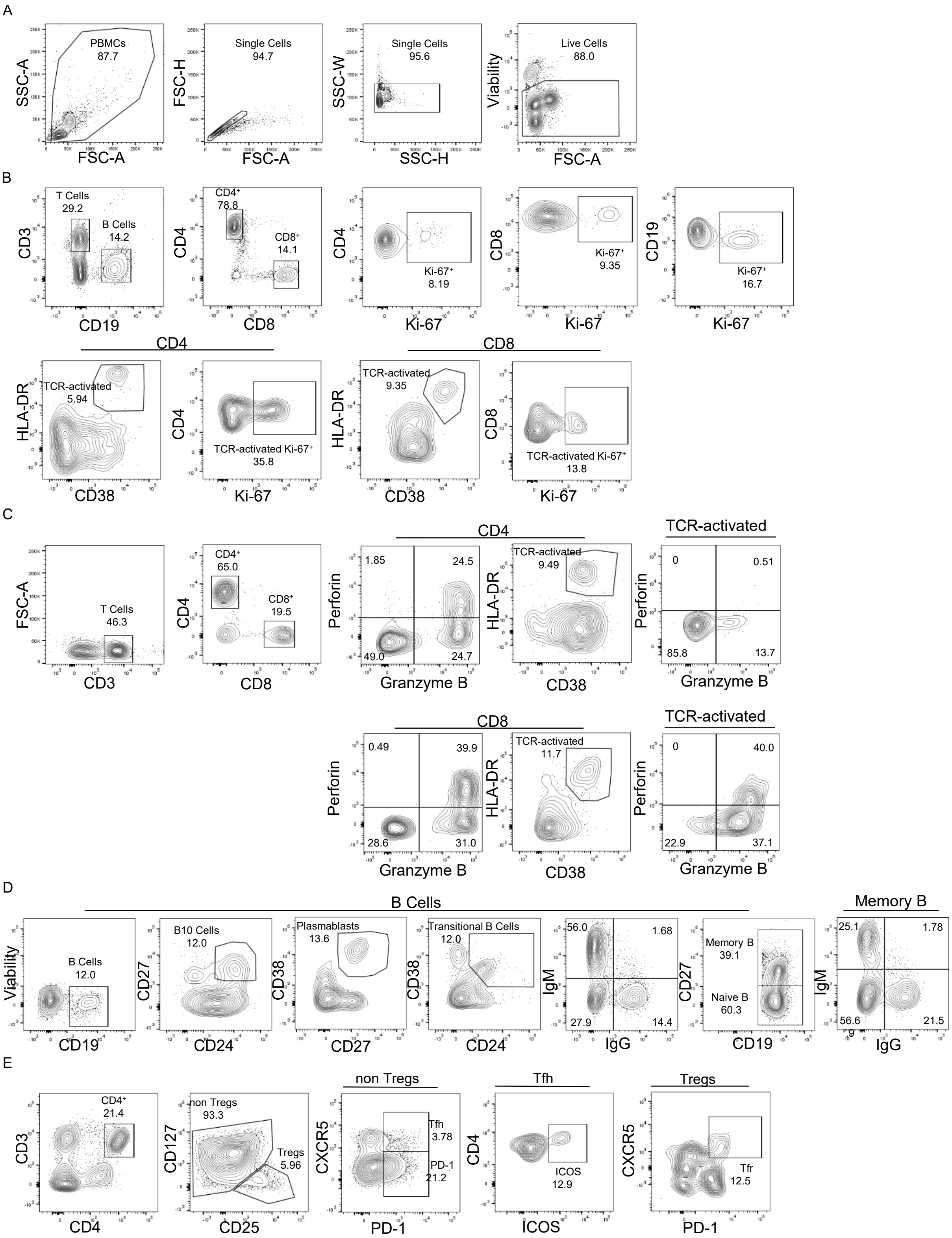
A
PBMCs 87.7
Single Cells 94.7
Single Cells 95.6
Live Cells 88.0
SSC-A
FSC-A
FSC-H
SSC-W
SSC-H
Viability
B
T Cells 29.2
B Cells 14.2
CD3
CD19
CD4+ 78.8
CD8+ 14.1
CD4
CD8
Ki-67+ 8.19
Ki-67+ 9.35
Ki-67+ 16.7
Ki-67
CD4
CD8
TCR-activated 5.94
HLA-DR
CD38
TCR-activated Ki-67+ 35.8
TCR-activated 9.35
TCR-activated Ki-67+ 13.8
C
T Cells 46.3
FSC-A
CD3
CD4+ 65.0
CD8+ 19.5
CD4
1.85
24.5
49.0
24.7
Perforin
Granzyme B
TCR-activated 9.49
TCR-activated
0
0.51
85.8
13.7
CD8
0.49
39.9
28.6
31.0
TCR-activated 11.7
0
40.0
22.9
37.1
D
B Cells
Memory B
B Cells 12.0
Viability
CD19
B10 Cells 12.0
CD27
CD24
Plasmablasts 13.6
CD38
Transitional B Cells 12.0
56.0
1.68
27.9
14.4
IgM
IgG
Memory B 39.1
Naive B 60.3
25.1
1.78
56.6
21.5
E
CD4+ 21.4
non Tregs 93.3
Tregs 5.96
CD127
CD25
non Tregs
Tfh 3.78
PD-1 21.2
CXCR5
PD-1
Tfh
ICOS 12.9
ICOS
Tregs
Tfr 12.5

F

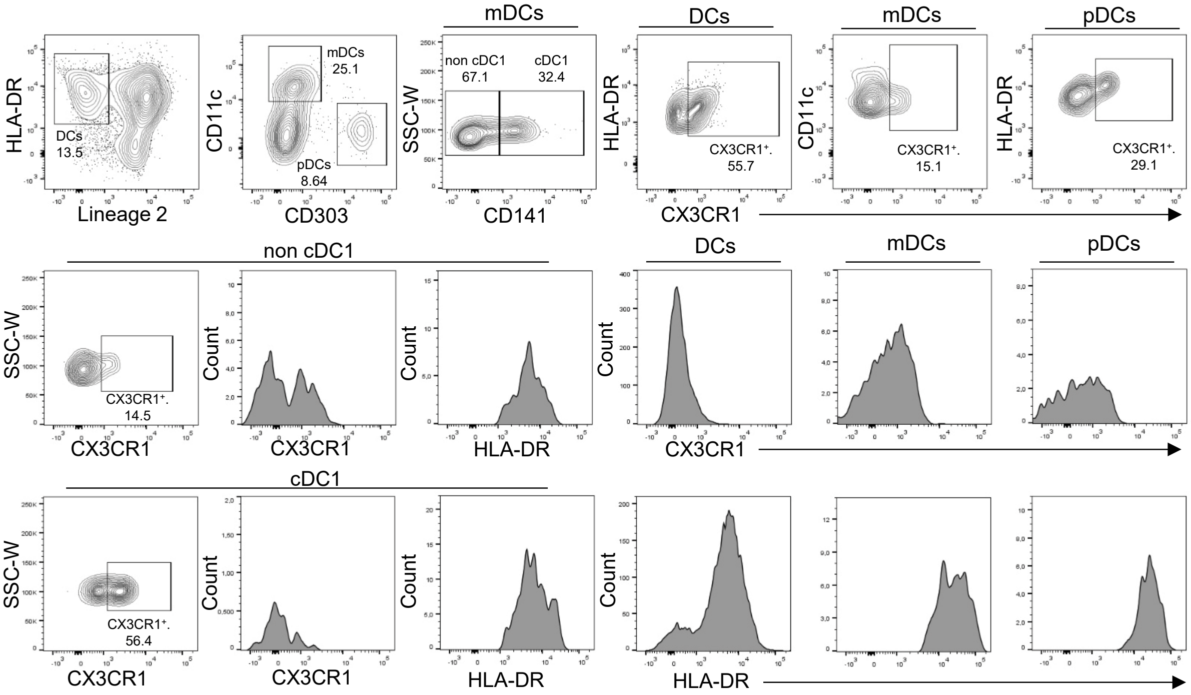
mDCs
DCs
mDCs
pDCs
HLA-DR
DCs
13.5
Lineage 2
CD11c
mDCs
25.1
pDCs
8.64
CD303
SSC-W
non cDC1
67.1
cDC1
32.4
CD141
CX3CR1⁺.
55.7
CX3CR1⁺.
15.1
CX3CR1⁺.
29.1
CX3CR1
non cDC1
DCs
mDCs
pDCs
CX3CR1⁺.
14.5
Count
HLA-DR
cDC1
CX3CR1⁺.
56.4

G

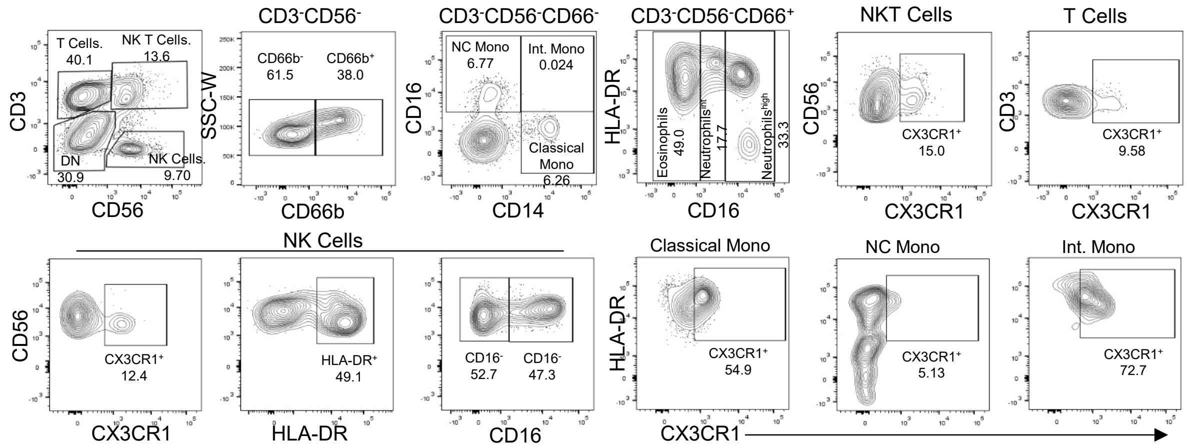
CD3⁻CD56⁻
CD3⁻CD56⁻CD66⁻
CD3⁻CD56⁻CD66⁺
NKT Cells
T Cells
T Cells.
40.1
NK T Cells.
13.6
DN
30.9
NK Cells.
9.70
CD3
CD56
CD66b⁻
61.5
CD66b⁺
38.0
SSC-W
CD66b
NC Mono
6.77
Int. Mono
0.024
Classical Mono
6.26
CD16
CD14
HLA-DR
Eosinophils
49.0
Neutrophils^int
17.7
Neutrophils^high
33.3
CX3CR1⁺
15.0
CX3CR1⁺
9.58
CX3CR1
NK Cells
Classical Mono
NC Mono
Int. Mono
CX3CR1⁺
12.4
HLA-DR⁺
49.1
CD16⁻
52.7
CD16⁻
47.3
CX3CR1⁺
54.9
CX3CR1⁺
5.13
CX3CR1⁺
72.7

H

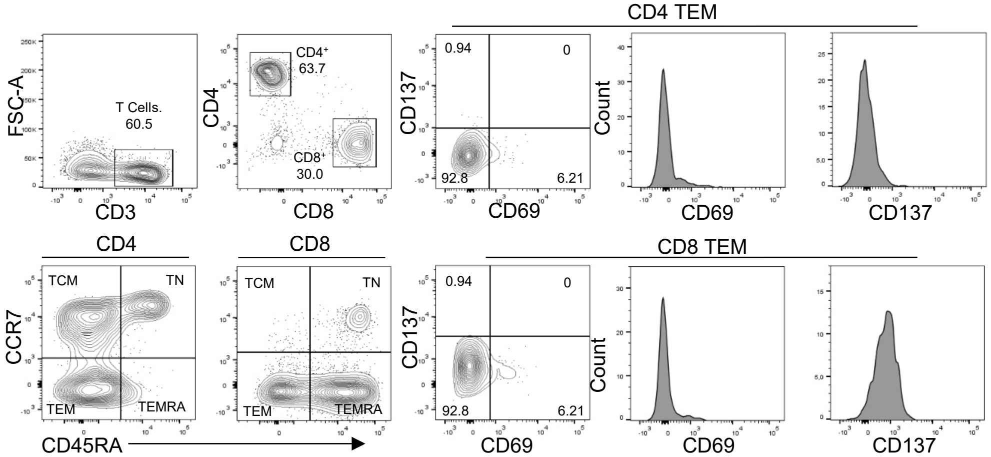
CD4 TEM
FSC-A
T Cells.
60.5
CD3
CD4
CD4⁺
63.7
CD8⁺
30.0
CD8
CD137
0.94
0
92.8
6.21
CD69
Count
CD4
CD8
CD8 TEM
CCR7
TCM
TN
TEM
TEMRA
CD45RA

Supplementary Figure 1. Gating strategies for the key cell populations described in Fig. 1-4, Fig. 6A-B, Fig. 7, and other supplementary figures. A, Gating strategy to identify all populations described in B-H. **B,** B and T cell surface staining gating strategy to identify B cells proliferation, CD4 and CD8 T cells, proliferation and TCR-activated T cells. **C,** T cell surface staining gating strategy to identify CD4 and CD8 T cells producing granzyme B and perforin in the population with or without TCR-activated T cells. **D,** B cell surface staining gating strategy to identify B10 cells, plasmablasts, transitional B cells, IgM cells, IgG cells, memory and naive cells populations. **E,** T cell surface staining gating strategy to identify Treg and TFH CD4 cells population. **F,** DCs surface staining gating strategy to identify differences in HLA-DR and CX3CR1 expression in DCs, mDC and pDCs cell populations. **G,** Innate cells and T surface staining gating strategy to identify NK cells, NK T cells, Eosinophils, Neutrophils and Monocytes populations. **H,** T cell surface staining gating strategy to identify differences in CD137 and CD69 expression in CD4 and CD8 T cells effector memory, central memory, terminally differentiated and naïve population.

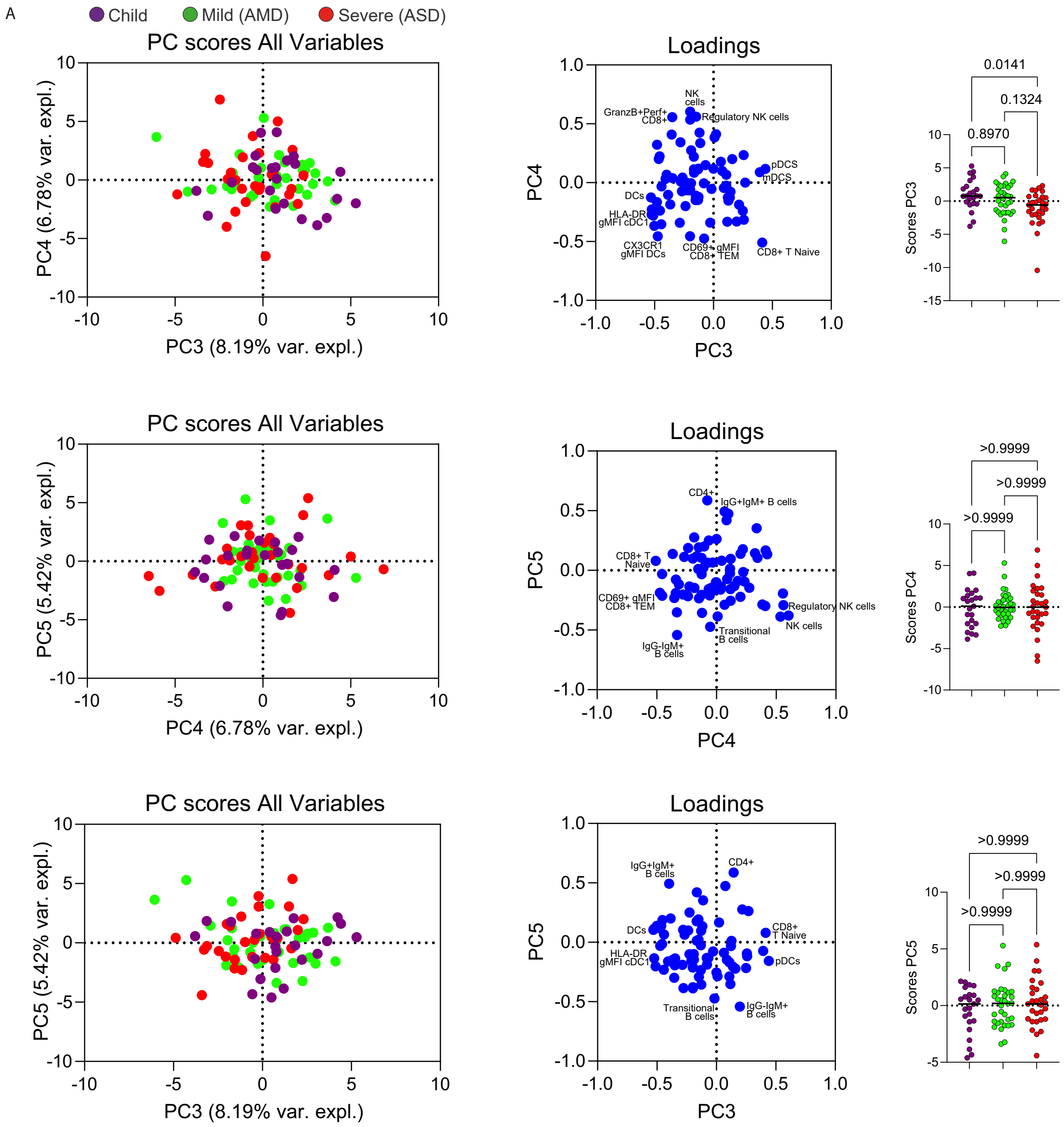

A
Child
Mild (AMD)
Severe (ASD)
PC scores All Variables
PC4 (6.78% var. expl.)
PC3 (8.19% var. expl.)
Loadings
PC4
PC3
NK cells
GranzB+Perf+ CD8+
Regulatory NK cells
pDCS
mDCS
DCs
HLA-DR gMFI cDC1
CX3CR1 gMFI DCs
CD69+ gMFI CD8+ TEM
CD8+ T Naive
Scores PC3
0.0141
0.1324
0.8970
PC scores All Variables
PC5 (5.42% var. expl.)
PC4 (6.78% var. expl.)
Loadings
PC5
PC4
CD4+
IgG+IgM+ B cells
CD8+ T Naive
CD69+ gMFI CD8+ TEM
Regulatory NK cells
NK cells
Transitional B cells
IgG-IgM+ B cells
Scores PC4
>0.9999
>0.9999
>0.9999
PC scores All Variables
PC5 (5.42% var. expl.)
PC3 (8.19% var. expl.)
Loadings
PC5
PC3
IgG+IgM+ B cells
CD4+
DCs
CD8+ T Naive
HLA-DR gMFI cDC1
pDCs
Transitional B cells
IgG-IgM+ B cells
Scores PC5
>0.9999
>0.9999
>0.9999

B

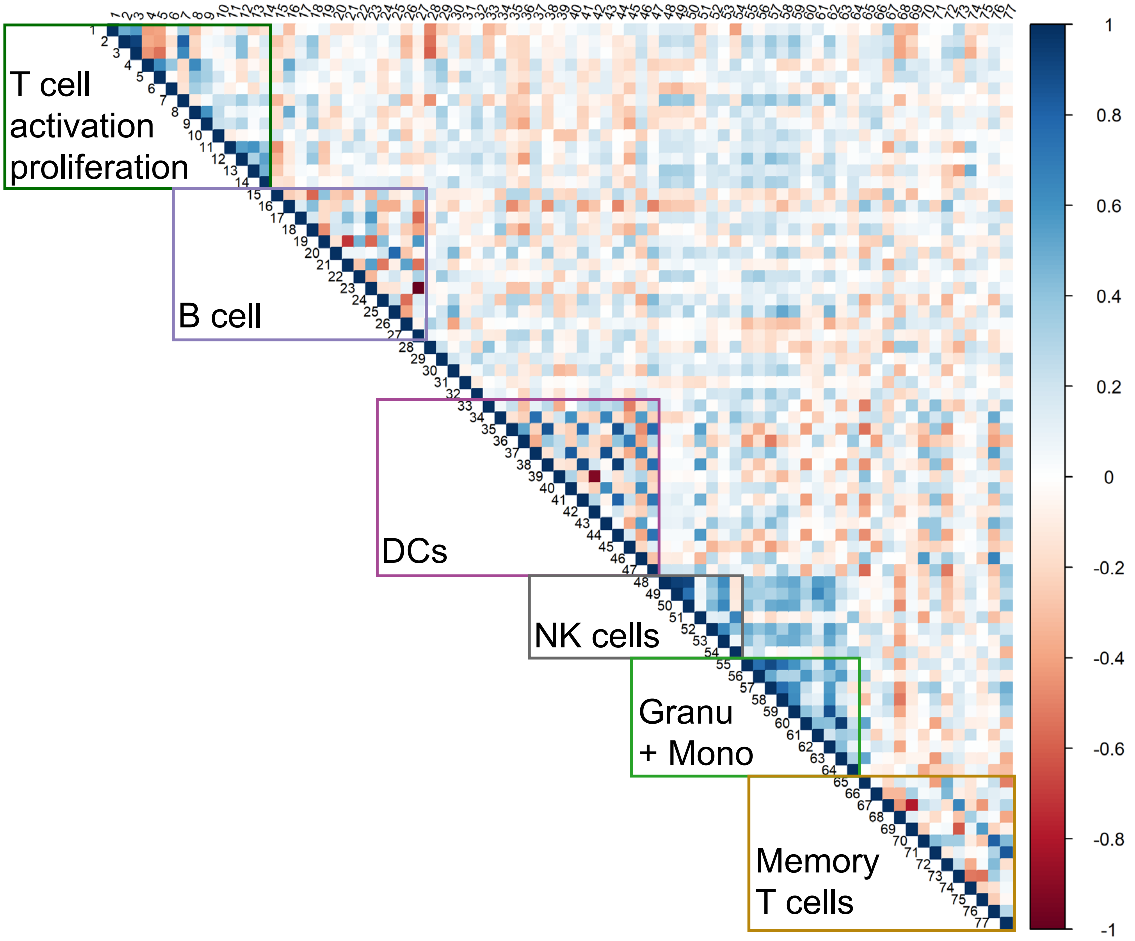

C

1	PBMCs	27	Naïve B Cells	53	HLA-DR$^+$ NK Cells
2	T cells	28	PD-1$^+$ CD4$^+$ T	54	CX3CR1$^+$ NK T cells
3	CD4$^+$	29	Tfh cells	55	CD66$^+$ Granulocytes
4	Ki-67$^+$ CD4$^+$	30	ICOS$^+$ Tfh cells	56	CD66b$^+$ CD16 hi Neutrophils
5	TCR-activated CD4$^+$	31	Treg	57	CD66b$^+$ CD16 int Neutrophils
6	TCR-activated Ki-67$^+$ CD4$^+$	32	CXCR5$^+$ Treg	58	CD66b$^+$ CD16 low Eosinophils
7	CD8$^+$	33	DCs	59	Classical Monocytes
8	Ki-67$^+$ CD8$^+$	34	CX3CR1 gMFI DCs	60	Intermediate Monocytes
9	TCR-activated CD8$^+$	35	HLA-DR gMFI DCs	61	NC Monocytes
10	TCR-activated Ki-67$^+$ CD8$^+$	36	mDCs	62	CX3CR1$^+$ Classical Monocytes
11	GranzB$^+$Perf$^+$ CD4$^+$	37	CX3CR1 gMFI mDCs	63	CX3CR1$^+$ Intermediate Monocytes
12	TCR-activated GranzB$^+$Perf$^+$ CD4$^+$	38	HLA-DR gMFI mDCs	64	CX3CR1$^+$ NC Monocytes
13	GranzB$^+$Perf$^+$ CD8$^+$	39	cDC1	65	CX3CR1$^+$ T cells
14	TCR-activated GranzB$^+$Perf$^+$ CD8$^+$	40	CX3CR1 gMFI cDC1	66	CD4$^+$ TCM
15	B cells	41	HLA-DR gMFI cDC1	67	CD4$^+$ T Naive
16	Ki-67$^+$ B cells	42	Non cDC1	68	CD4$^+$ TEMRA
17	B10 cells	43	CX3CR1 gMFI Non cDC1	69	CD4$^+$ TEM
18	Plasmablasts	44	HLA-DR gMFI Non cDC1	70	CD69$^+$ gMFI CD4$^+$ TEM
19	IgG$^-$IgM$^+$ B cells	45	pDCs	71	CD137$^+$ gMFI CD4$^+$ TEM
20	IgG$^+$IgM$^+$ B cells	46	CX3CR1 gMFI pDCs	72	CD8$^+$ TCM
21	IgG$^+$IgM- B cells	47	HLA-DR gMFI pDCs	73	CD8$^+$ T Naive
22	Transitional B cells	48	NK cells	74	CD8$^+$ TEMRA
23	Memory B cells	49	CD16$^+$NK cells	75	CD8$^+$ TEM
24	IgG$^-$IgM$^+$ Memory B cells	50	CD16$^-$ NK cells	76	CD69$^+$ gMFI CD8$^+$ TEM
25	IgG$^+$IgM$^+$ Memory B cells	51	NK T cells	77	CD137$^+$ gMFI CD8$^+$ TEM
26	IgG$^+$IgM$^-$ Memory B cells	52	CX3CR1$^+$ NK Cells		

Supplementary Figure 2. Principal component analysis and spearman correlation of all variables composing the immune profile. A, Principal component analysis of the distribution of clusters using additional PCs (PC3, PC4 and PC5). Variable contribution is also shown, where each blut dot represents a variable and the major ones are highlighted. Comparison of scores for each PC by analysis of variance (Kruskal-Wallis), horizontal bars represent median. **B**, Clusters of more correlated variables are outlined and identified in a Spearman correlation matrix of all variables. **C**, List of immune variables indicated by numbers by which they are plotted on the matrix. Source data are provided as a Source Data file.

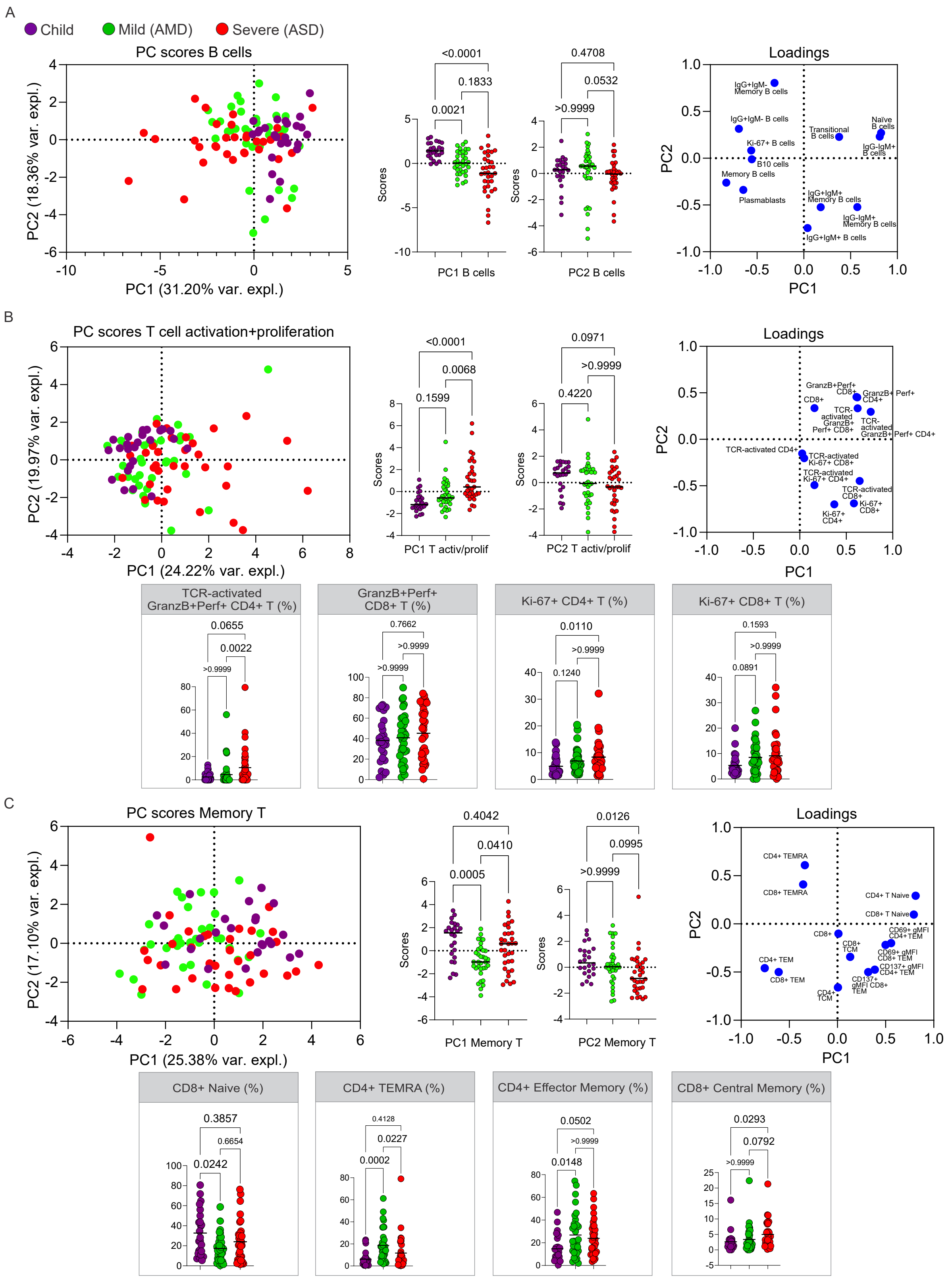

Supplementary Figure 3. Principal Component Analysis of adaptive cells immune signatures. A, B cells; **B**, Proliferating/activated T cells; and **C**, Memory T cells). For each signature, are displayed the PCA plot of PC1xPC2, the differences in scores of individuals for each PC; the loadings of the main variables contributing to each PC; and Kruskal-Wallis tests comparisons of the major contributing variables values for each group of patients. Horizontal bars on PC scores represent median, and on percentage of cells mean. Source data are provided as a Source Data file.

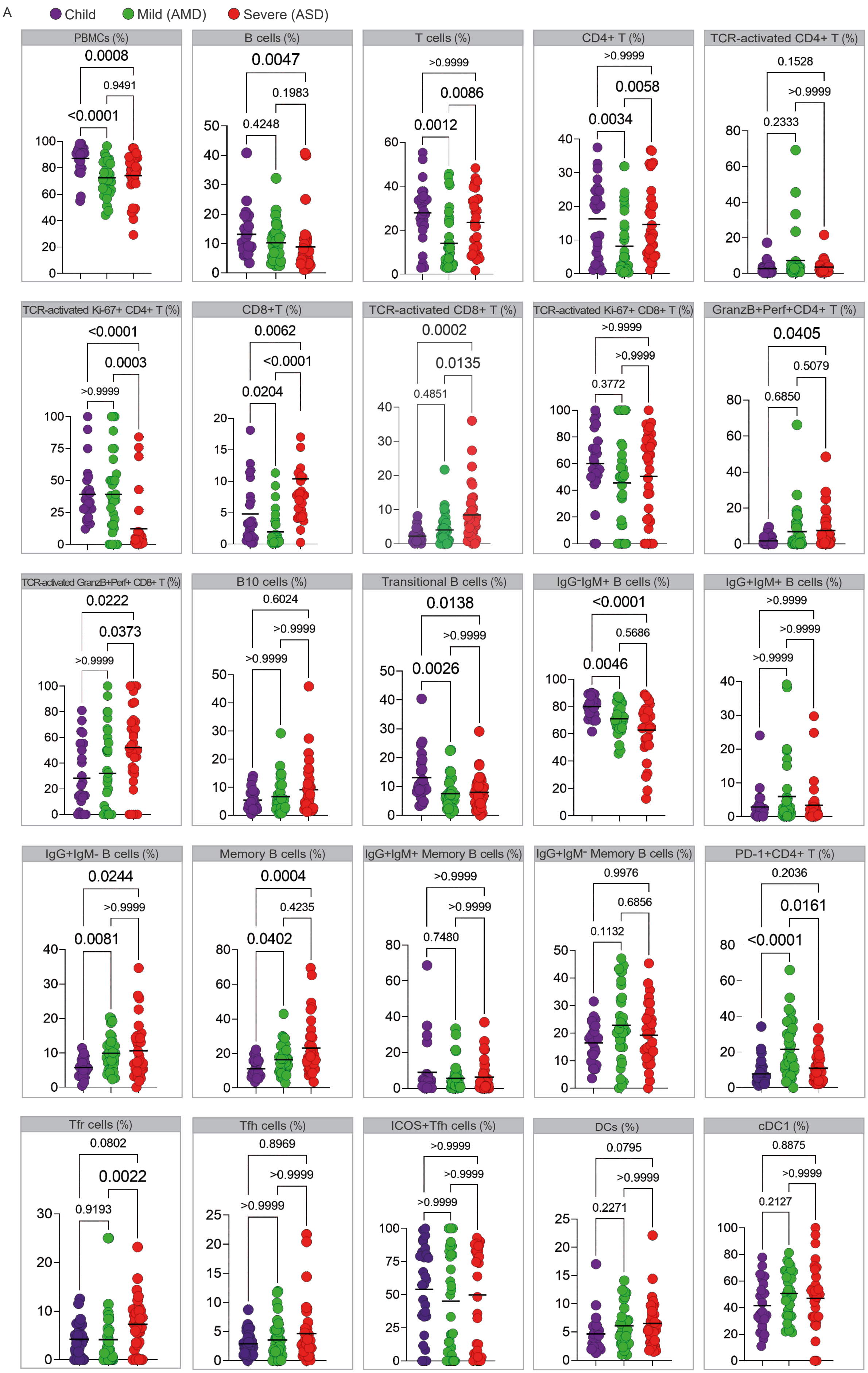

A
Child
Mild (AMD)
Severe (ASD)
PBMCs (%)
0.0008
0.9491
<0.0001
B cells (%)
0.0047
0.1983
0.4248
T cells (%)
>0.9999
0.0086
0.0012
CD4+ T (%)
>0.9999
0.0058
0.0034
TCR-activated CD4+ T (%)
0.1528
>0.9999
0.2333
TCR-activated Ki-67+ CD4+ T (%)
<0.0001
0.0003
>0.9999
CD8+T (%)
0.0062
<0.0001
0.0204
TCR-activated CD8+ T (%)
0.0002
0.0135
0.4851
TCR-activated Ki-67+ CD8+ T (%)
>0.9999
>0.9999
0.3772
GranzB+Perf+CD4+ T (%)
0.0405
0.5079
0.6850
TCR-activated GranzB+Perf+ CD8+ T (%)
0.0222
0.0373
>0.9999
B10 cells (%)
0.6024
>0.9999
>0.9999
Transitional B cells (%)
0.0138
>0.9999
0.0026
IgG-IgM+ B cells (%)
<0.0001
0.5686
0.0046
IgG+IgM+ B cells (%)
>0.9999
>0.9999
>0.9999
IgG+IgM- B cells (%)
0.0244
>0.9999
0.0081
Memory B cells (%)
0.0004
0.4235
0.0402
IgG+IgM+ Memory B cells (%)
>0.9999
>0.9999
0.7480
IgG+IgM- Memory B cells (%)
0.9976
0.6856
0.1132
PD-1+CD4+ T (%)
0.2036
0.0161
<0.0001
Tfr cells (%)
0.0802
0.0022
0.9193
Tfh cells (%)
0.8969
>0.9999
>0.9999
ICOS+Tfh cells (%)
>0.9999
>0.9999
>0.9999
DCs (%)
0.0795
>0.9999
0.2271
cDC1 (%)
0.8875
>0.9999
0.2127

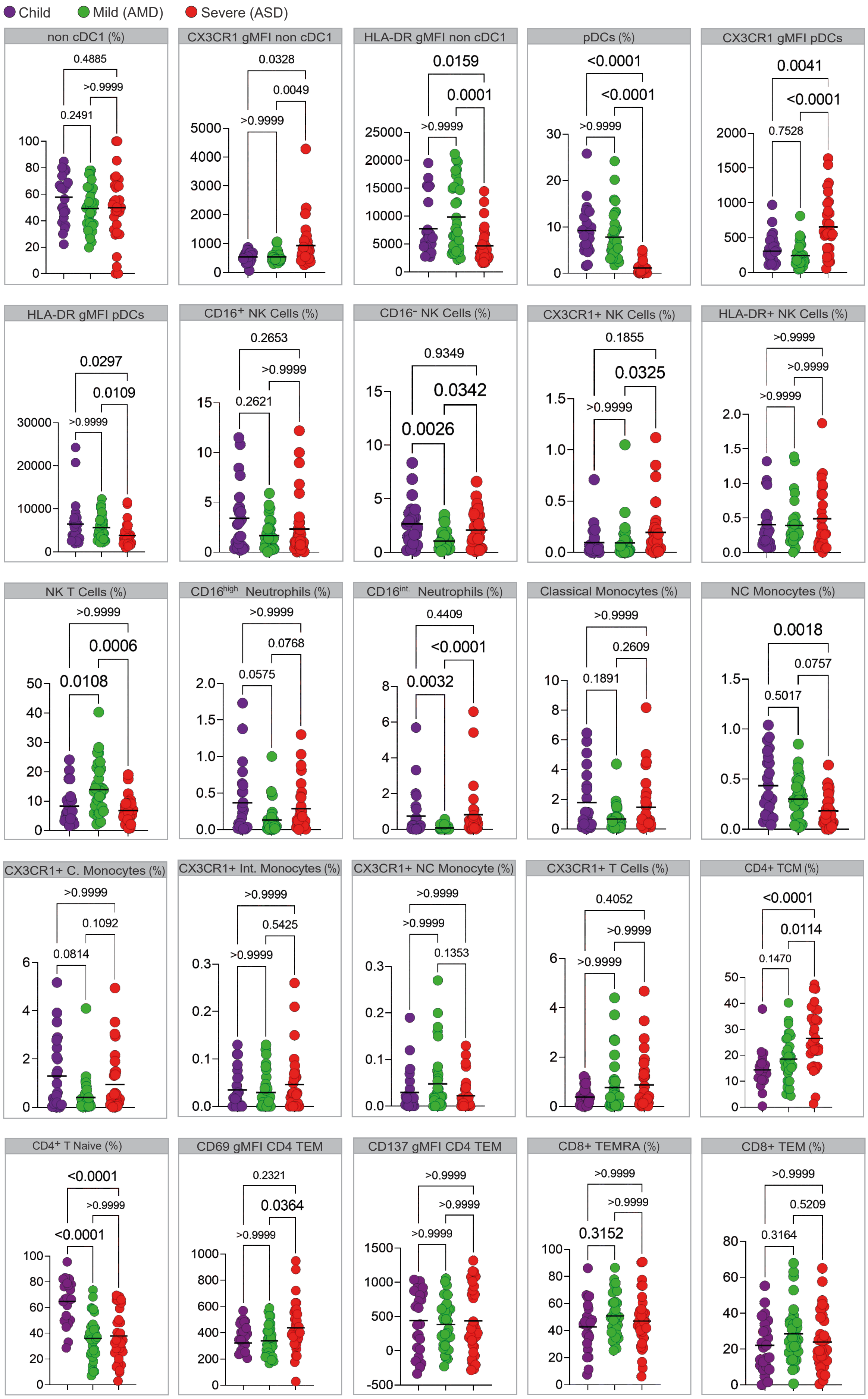

Child
Mild (AMD)
Severe (ASD)
non cDC1 (%)
0.4885
>0.9999
0.2491
CX3CR1 gMFI non cDC1
0.0328
0.0049
>0.9999
HLA-DR gMFI non cDC1
0.0159
0.0001
>0.9999
pDCs (%)
<0.0001
<0.0001
>0.9999
CX3CR1 gMFI pDCs
0.0041
<0.0001
0.7528
HLA-DR gMFI pDCs
0.0297
0.0109
>0.9999
CD16+ NK Cells (%)
0.2653
>0.9999
0.2621
CD16- NK Cells (%)
0.9349
0.0342
0.0026
CX3CR1+ NK Cells (%)
0.1855
0.0325
>0.9999
HLA-DR+ NK Cells (%)
>0.9999
>0.9999
>0.9999
NK T Cells (%)
>0.9999
0.0006
0.0108
CD16high Neutrophils (%)
>0.9999
0.0768
0.0575
CD16int. Neutrophils (%)
0.4409
<0.0001
0.0032
Classical Monocytes (%)
>0.9999
0.2609
0.1891
NC Monocytes (%)
0.0018
0.0757
0.5017
CX3CR1+ C. Monocytes (%)
>0.9999
0.1092
0.0814
CX3CR1+ Int. Monocytes (%)
>0.9999
0.5425
>0.9999
CX3CR1+ NC Monocyte (%)
>0.9999
>0.9999
0.1353
CX3CR1+ T Cells (%)
0.4052
>0.9999
>0.9999
CD4+ TCM (%)
<0.0001
0.0114
0.1470
CD4+ T Naive (%)
<0.0001
>0.9999
<0.0001
CD69 gMFI CD4 TEM
0.2321
0.0364
>0.9999
CD137 gMFI CD4 TEM
>0.9999
>0.9999
>0.9999
CD8+ TEMRA (%)
>0.9999
>0.9999
0.3152
CD8+ TEM (%)
>0.9999
0.5209
0.3164

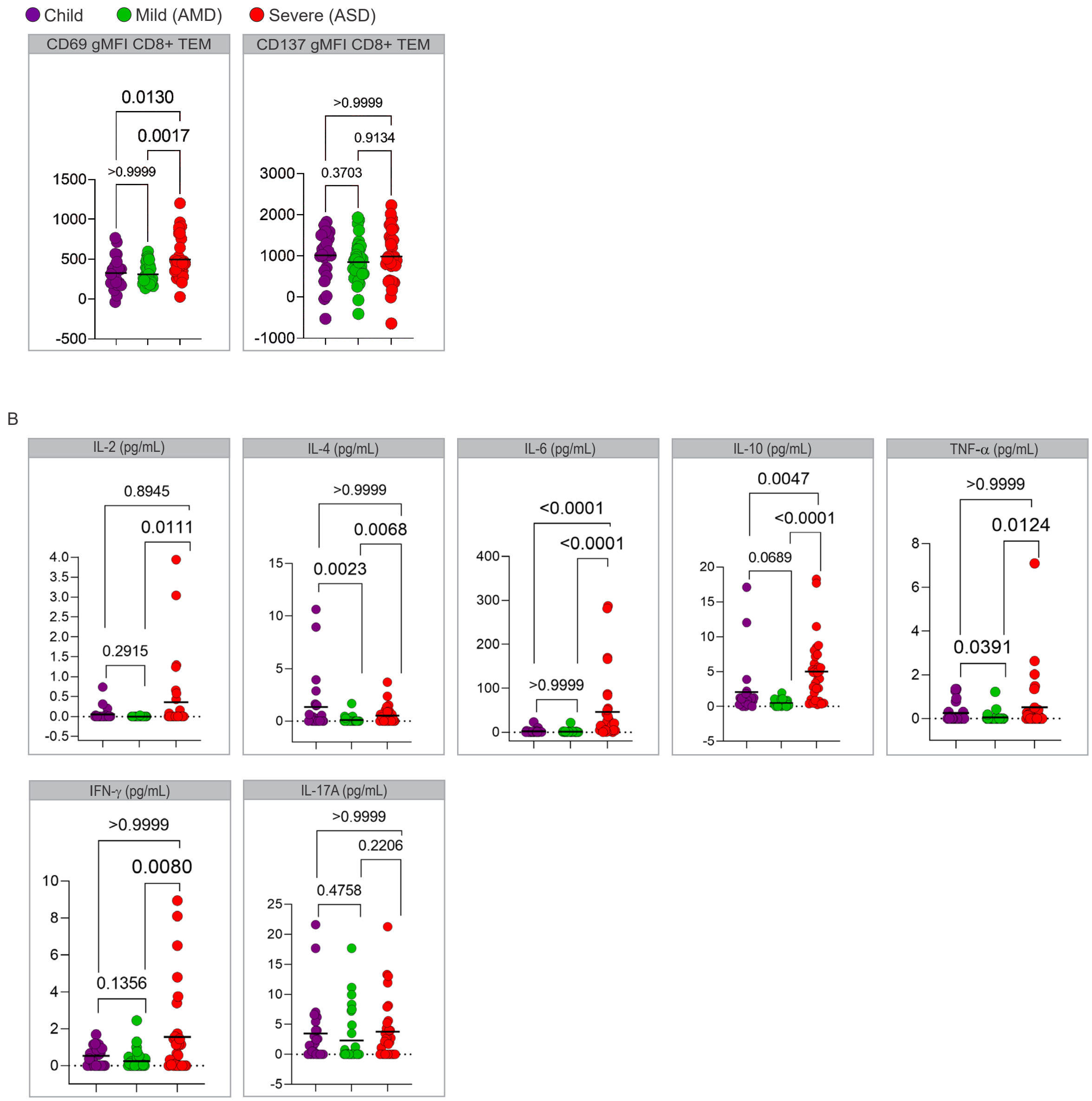

Supplementary Figure 4. Analysis of variance (Kruskal-Wallis) of remaining immune variables and cytokine expression. A, Analysis of variance of the values for immune variables (in percentages or gMFI) that were lesser influencers on the first two principal components and thus not included in the main figures. **B**, cytokine analysis measured from patients plasma. Horizontal bars represent mean. Source data are provided as a Source Data file.

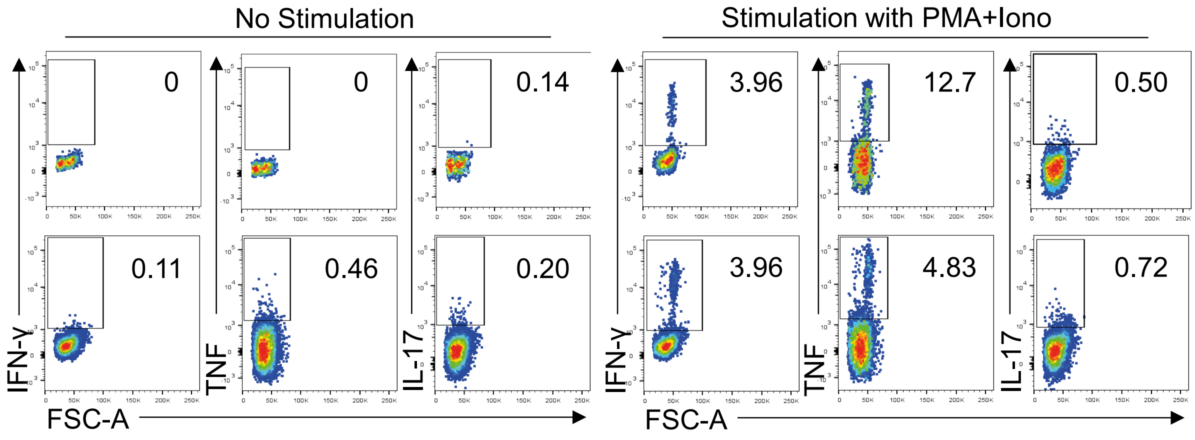

Supplementary Figure 5. Control gate strategies for flow cytometry analysis of specific T cell responses. Negative (DMSO) and positive (PMA+Ionomycin) controls gate strategies and representative plots of CD4+ or CD8+ T cell simulations.

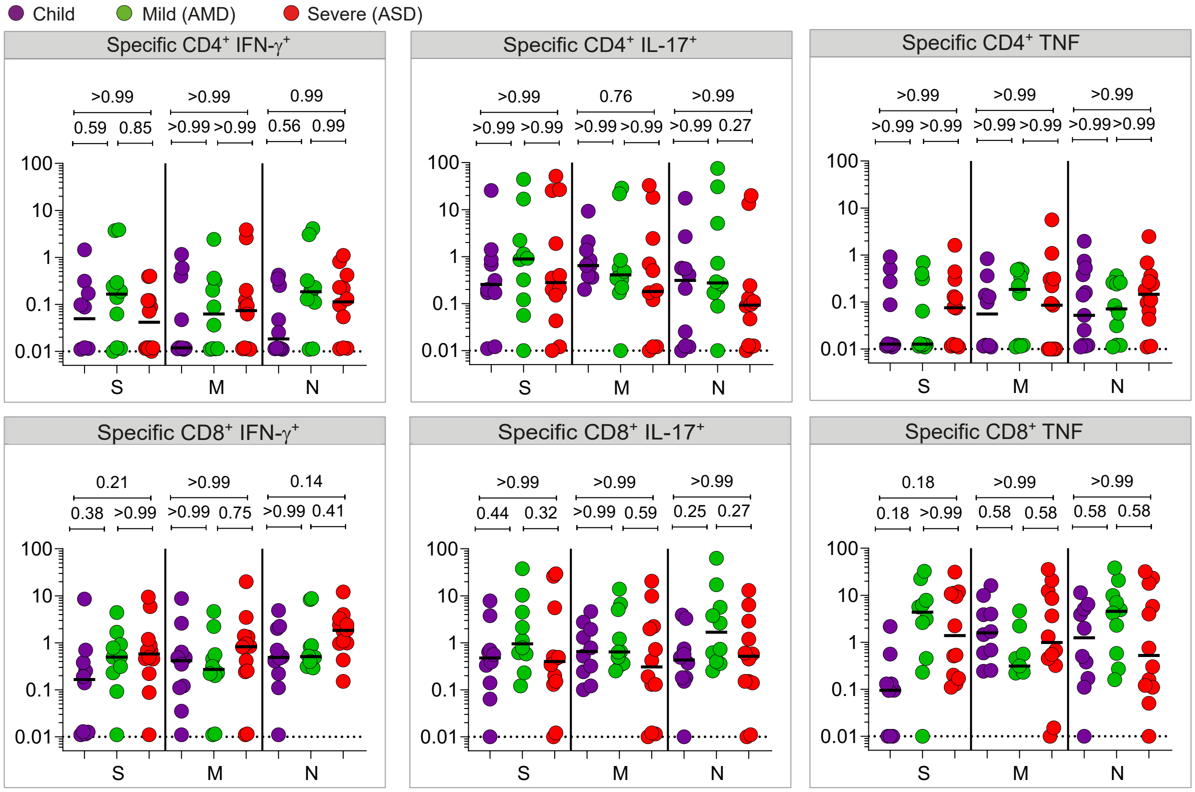

Supplementary Figure 6. Comparison of specific T cell responses by effector T cell type among the groups. Values of specific T cell responses (in percentages of positive CD4+ and CD8+ positive cytokine expressing cells in response to peptide pools) are plotted. Each dot represents a patient, color-coded: children – purple, adult with mild disease – green, and adult with severe disease – red. All analyses are Kruskal-Wallis tests, and the p values are indicated in brackets. Horizontal bars represent median. Source data are provided as a Source Data file.

Supplementary Table 1. Principal Component Analysis (PCA) variances and loading.

**PCA all variables**								
Positive (loadings)	**PC1**	1	2	3	**PC2**	1	2	3
		Memory B IgG$^-$IgM$^+$	Naïve B cells	HLA-DR gMFI cDC1		Treg	mDC	CD4$^+$ TEMRA
		0.57	0.56	0.55		0.53	0.45	0.40
Negative (loadings)	**PC1**	1	2	3	**PC2**	1	2	3
		B cells Ki67$^+$	Plasma blasts	CX3CR1 gMFI DC		Eosino phils	NK cells	Granulo cytes
		-0.71	-0.58	-0.60		-0.58	-0.55	-0.48
Positive (loadings)	**PC3**	1	2	3	**PC4**	1	2	3
		pDCS	CD8$^+$ T Naïve	mDC		NK cells	NK Regulatory	GranzB$^+$ Perf$^+$CD8$^+$
		0.44	0.41	0.39		0.60	0.56	0.56
Negative (loadings)	**PC3**	1	2	3	**PC4**	1	2	3
		DCs	HLA-DR gMFI cDC1	CD66$^+$ Granu		CD69$^+$ gMFI CD8$^+$TEM	CX3CR1 gMFI DCs	CD69$^+$ gMFI CD4$^+$TEM
		-0.53	-0.53	-0.52		-0.48	-0.46	-0.46
Positive (loadings)	**PC5**	1	2	3				
		CD4+	IgG$^+$IgM$^+$ B cells	T cells				
		0.59	0.49	0.47				
Negative (loadings)	**PC5**	1	2	3				
		IgG$^-$IgM$^+$ B cells	transitional B cells	Cytotoxic NK cells				
		-0.54	-0.47	-0.39				
**PCA Granu+Mono**								
Positive (loadings)	**PC1**	1	2	3	**PC2**	1	2	
		Classical Monocytes	Inter-mediate Monocytes	CX3CR1$^+$ Inter-mediate Monocytes		CX3CR1$^+$ Classical Monocytes	CD16 Int Neutrophils	
		0.84	0.80	0.75		0.43	0.37	
Negative (loadings)	**PC1**	1	2	3	**PC2**	1	2	
		-	-	-		NC Monocytes	CX3CR1$^+$ NC Monocytes	
		-	-	-		-0.66	-0.64	
**PCA NK cells**								
Positive (loadings)	**PC1**	1	2	3	**PC2**	1	2	
		HLA-DR$^+$ NK Cells	Regulatory NK cells	CX3CR1$^+$ NK		CX3CR1$^+$NK T	NK T cells	
		0.85	0.77	0.73		0.85	0.69	
Negative (loadings)	**PC1**	1	2	3	**PC2**	1	2	
		-	-	-		Cytotoxic NK cells	Regulatory NK cells	
		-	-	-		-0.43	-0.34	
**PCA Dendritic Cells**								
Positive (loadings)	**PC1**	1	2	3	**PC2**	1	2	3
		CX3CR1 gMFI mDCs	CX3CR1 gMFI cDC1	CX3CR1 gMFI Non cDC1		pDCs	mDCs	cDC1
		0.57	0.51	0.44		0.59	0.57	0.23
	**PC1**	1	2	3	**PC2**	1	2	3

Negative (loadings)		HLA-DR gMFI mDCs	HLA-DR gMFI DCs	HLA-DR gMFI cDC1		CX3CR1 gMFI mDCs	CX3CR1 gMFI cDC1	CX3CR1 gMFI Non cDC1
		-0.85	-0.85	-0.83		-0.70	-0.60	-0.53
**PCA T cell activation + proliferation**								
Positive (loadings)	**PC1**	1	2	3	**PC2**	1	2	
		TCR-activated GranzB^{+} Perf^{+}CD4^{+}	TCR-activated CD8^{+}	TCR-activated GranzB^{+} Perf^{+}CD8^{+}		GranzB^{+} Perf^{+}CD8^{+}	GranzB^{+} Perf^{+}CD4^{+}	
		0.76	0.64	0.62		0.45	0.45	
Negative (loadings)	**PC1**	1	2	3	**PC2**	1	2	
		-	-	-		Ki67^{+} CD4	Ki67^{+} CD8	
		-	-	-		-0.70	-0.69	
**PCA B cells**								
Positive (loadings)	**PC1**	1	2	3	**PC2**	1	2	
		IgG^{-}IgM^{+} B cells	Naïve B Cells	IgG^{-}IgM^{+} Memory B cells		IgG^{+} IgM^{-} Memory B cells	-	
		0.82	0.69	0.67		0.75	-	
Negative (loadings)	**PC1**	1	2	3	**PC2**	1	2	
		IgG^{+} IgM^{-} B cells	Plasma blasts	Ki-67^{+} B cells		IgG^{+} IgM^{+} B cells	IgG^{+}IgM^{+} Memory B cells	
		-0.75	-0.67	-0.59		-0.80	-0.57	
**PCA T cell memory**								
Positive (loadings)	**PC1**	1	2		**PC2**	1	2	
		CD4^{+} T Naïve	CD8^{+} T Naïve			CD4^{+} TEMRA	CD8^{+} TEMRA	
		0.81	0.79			0.61	0.41	
Negative (loadings)	**PC1**	1	2		**PC2**	1	2	
		CD4^{+} TEM	CD8^{+} TEM			CD4^{+} TCM	CD8^{+} TEM	
		-0.76	-0.61			-0.66	-0.50	

Supplementary Table 2. Antibody information.

Antibody	Cat.	Aplication	Dilution
CD3 APC-H7	641397	Flow Cytometry	1:5
CD24 APC-H7	658331	Flow Cytometry	1:20
HLA-DR APC-H7	561358	Flow Cytometry	1:20
CD4 PerCP	560650	Flow Cytometry	1:20
CD27 PerCP	560612	Flow Cytometry	1:20
CD11c PerCP	565227	Flow Cytometry	1:20
CD14 PerCP	550787	Flow Cytometry	1:20
CD8 FITC	555634	Flow Cytometry	1:5
IgG FITC	555786	Flow Cytometry	1:5
Lineage2 FITC	643397	Flow Cytometry	1:5
CD16 FITC	555406	Flow Cytometry	1:5
CXCR5 (CD185) BB515	564624	Flow Cytometry	1:20
CD19 APC	555415	Flow Cytometry	1:5
CD127 Alexa 647	558598	Flow Cytometry	1:5
CX3CR1 Alexa 647	565895	Flow Cytometry	1:20
CD69 APC	560711	Flow Cytometry	1:20
CD38 PE	555460	Flow Cytometry	1:5
ICOS (CD278) PE	557802	Flow Cytometry	1:5
CD141 PE	559781	Flow Cytometry	1:5
CD66b PE	561650	Flow Cytometry	1:20
CD137 (4-1BB) PE	555956	Flow Cytometry	1:5
CD19 PE-Cy7	557835	Flow Cytometry	1:20
CD25 PE-Cy7	335789	Flow Cytometry	1:20
CD45RA PE-Cy7	337167	Flow Cytometry	1:20
HLA-DR PE-Cy7	560651	Flow Cytometry	1:20
CD56 BV421	562751	Flow Cytometry	1:20
CD303 BV421	566427	Flow Cytometry	1:20
CCR7 (CD197) BV421	566743	Flow Cytometry	1:20
Ki-67 BV421	562899	Flow Cytometry	1:20
IgM BV421	562618	Flow Cytometry	1:20
PD-1 (CD279) BV421	564323	Flow Cytometry	1:20
Perforina Alexa 647	563576	Flow Cytometry	1:20
Granzima B BV421	563389	Flow Cytometry	1:20
CD3 PE-Cy7	557851	Flow Cytometry	1:20
CD8 APC-H7	560179	Flow Cytometry	1:20
IFN-y FITC	554551	Flow Cytometry	1: 100
TNF APC	551384	Flow Cytometry	1: 30
IL-17A PE	560436	Flow Cytometry	1:5
Fixable Viability VS510	564406	Flow Cytometry	1:1.000
IgG HRP	IC-1H01	ELISA	1:10.000
IgA HRP	A18781	ELISA	1:10.000

Supplementary Note 1. Consortium members

COVIDa study group: Luciane Beatriz Kern, Thaís Raupp Azevedo, Maristênia Machado Araújo, Amanda Paz Santos, Shirlei Villanova Ribeiro, Fernando Rovedder Boita, Camila Dietrich, Fernanda Lutz Tolves, Jaina da Costa Pereira, Adriana Isabel Rohden, Thainá Dias Luft, Shirlei Villanova Ribeiro, Catia Moreira Guterres, Caroline Cabral Robinson, Débora Vacaro Fogazzi, Regis Goulart Rosa, Ana Paula dos Santos, Gisele Alcina Nader Bastos, Denise Arakaki-Sanchez, Maicon Falavigna, Patricia Bartholomay Oliveira e Francieli Fontana Sutile Tardetti Fantinato.
