# Supplementary material for: Pediatric COVID-19 patients in South Brazil show abundant viral mRNA and strong specific anti-viral responses
Source: Nat Commun. 2021 Nov 25;12:6844. doi: 10.1038/s41467-021-27120-y (PMC8617275; doi:10.1038/s41467-021-27120-y)
Supplement: Supplementary file 3 — Reporting Summary [file 41467_2021_27120_MOESM3_ESM.pdf]

nature research

Corresponding author(s): Cristina Bonorino

Last updated by author(s): 10-29-2021

Reporting Summary

Nature Research wishes to improve the reproducibility of the work that we publish. This form provides structure for consistency and transparency in reporting. For further information on Nature Research policies, see our Editorial Policies and the Editorial Policy Checklist.

Statistics

For all statistical analyses, confirm that the following items are present in the figure legend, table legend, main text, or Methods section.

n/a	Confirmed	
☐	☒	The exact sample size (n) for each experimental group/condition, given as a discrete number and unit of measurement
☐	☒	A statement on whether measurements were taken from distinct samples or whether the same sample was measured repeatedly
☐	☒	The statistical test(s) used AND whether they are one- or two-sided<br>*Only common tests should be described solely by name; describe more complex techniques in the Methods section.*
☒	☐	A description of all covariates tested
☐	☒	A description of any assumptions or corrections, such as tests of normality and adjustment for multiple comparisons
☐	☒	A full description of the statistical parameters including central tendency (e.g. means) or other basic estimates (e.g. regression coefficient) AND variation (e.g. standard deviation) or associated estimates of uncertainty (e.g. confidence intervals)
☐	☒	For null hypothesis testing, the test statistic (e.g. F, t, r) with confidence intervals, effect sizes, degrees of freedom and P value noted<br>*Give P values as exact values whenever suitable.*
☒	☐	For Bayesian analysis, information on the choice of priors and Markov chain Monte Carlo settings
☒	☐	For hierarchical and complex designs, identification of the appropriate level for tests and full reporting of outcomes
☒	☐	Estimates of effect sizes (e.g. Cohen's d, Pearson's r), indicating how they were calculated

Our web collection on statistics for biologists contains articles on many of the points above.

Software and code

Policy information about availability of computer code

Data collection	Flow cytometry data was collected using a FACS-Canto II Becton Dickinson; the acquisition software was FACSDiva v.9.0. RT-PCR data was collected using QuantStudioTM5 Real-Time PCR System (Thermo-Fischer, with proprietary software).
Data analysis	Flow cytometry data analysis was performed using FlowJo V10.7.1 (Becton Dickinson). Statistics were performed either using GraphPad Prism, V.9. RT-PCR data was collected analyzed with Design and Analysis Software v2.3 (Thermo-Fischer).Spearman correlation analysis was performed on R ver. 4.0.3 using function corrplot. Principal components were extracted from raw data analyzed in FlowJo and later in GraphPad Prism. Three dimentional plotting was performed with python 2.7 using matplotlib version 1.5.1. CBA analisys performed with FCAP Array software 3.0.

For manuscripts utilizing custom algorithms or software that are central to the research but not yet described in published literature, software must be made available to editors and reviewers. We strongly encourage code deposition in a community repository (e.g. GitHub). See the Nature Research guidelines for submitting code & software for further information.

Data

Policy information about availability of data

All manuscripts must include a data availability statement. This statement should provide the following information, where applicable:

- Accession codes, unique identifiers, or web links for publicly available datasets
- A list of figures that have associated raw data
- A description of any restrictions on data availability

The data supporting the findings of this study are available within the article and its Supplementary Information files or from the corresponding authors on reasonable request.

Field-specific reporting

Please select the one below that is the best fit for your research. If you are not sure, read the appropriate sections before making your selection.

- [x] Life sciences
- [ ] Behavioural & social sciences
- [ ] Ecological, evolutionary & environmental sciences

For a reference copy of the document with all sections, see nature.com/documents/nr-reporting-summary-flat.pdf

Life sciences study design

All studies must disclose on these points even when the disclosure is negative.

Sample size	A prospective cohort study was carried out at Hospital Moinhos de Vento and at Hospital Restinga e Extremo Sul, both in Porto Alegre, southern Brazil. A convenience sample of adults and children older than 2 months were enrolled from June to December 2020 at either the outpatient clinics (OPC), emergency rooms (ER), or hospitalized.We have recruited a total of 92 patients (25 children; 34 adults with mild disease - AMD; and 33 adults with severe disease - ASD). All subjects had COVID-19 confirmed by PCR.
Data exclusions	No raw data was excluded from the analysis. For PCA analysis, redundant variables were excluded when they did not add information, as that is stated in the manuscript.
Replication	RNA extracted by nasal swabs is still available for replications. From blood samples, plasma is still available for replication, but few patients still have cryopreserved cells left. When cells were available, tests were performed twice. Due to limited amount of blood obtained by some individuals, their tests were performed only once. All attempts at replication were successful.
Randomization	This was an observational study to generate hypotheses, and not of a randomized, experimental design (as in testing drug effects, for example). Thus, randomization was not applicable.
Blinding	This was an observational study to generate hypotheses, and not of a randomized, experimental design (as in testing drug effects, for example). Thus, blinding was not applicable.

Reporting for specific materials, systems and methods

We require information from authors about some types of materials, experimental systems and methods used in many studies. Here, indicate whether each material, system or method listed is relevant to your study. If you are not sure if a list item applies to your research, read the appropriate section before selecting a response.

Materials & experimental systems

n/a	Involved in the study	
[ ]	[x]	Antibodies
[x]	[ ]	Eukaryotic cell lines
[x]	[ ]	Palaeontology and archaeology
[x]	[ ]	Animals and other organisms
[ ]	[x]	Human research participants
[x]	[ ]	Clinical data
[x]	[ ]	Dual use research of concern

Methods

n/a	Involved in the study	
[x]	[ ]	ChIP-seq
[ ]	[x]	Flow cytometry
[x]	[ ]	MRI-based neuroimaging

Antibodies

Antibodies used

APC-H7 CD3 BD Biosciences SK7 cat. 641397 lot. 241507 (Dilution 1:5)
APC-H7 CD24 BD Biosciences ML5 cat. 658331 lot. 99843 (Dilution 1:20)
APC-H7 HLA-DR BD Biosciences G46-6 cat. 561358 lot. 23290 (Dilution 1:20)
PerCP-Cy5.5 CD4 BD Biosciences RPA-T4 cat. 560650 lot. 9304627 (Dilution 1:20)
PerCP-Cy5.5 CD27 BD Biosciences M-T271 cat. 560612 lot. 44507 (Dilution 1:20)
PerCP-Cy5.5 CD11c BD Biosciences B-ly6 cat. 565227 lot. 9365348 (Dilution 1:20)
PerCP-Cy5.5 CD14 BD Biosciences M5E2 cat. 550787 lot. 22797 (Dilution 1:20)
FITC CD8 BD Biosciences HIT8a cat. 555634 lot. 265651 (Dilution 1:5)
FITC IgG BD Biosciences G18-145 cat. 555786 lot. 6092901 (Dilution 1:5)
FITC Lineage 2 BD Biosciences - cat. 643397 lot. 260802 (Dilution 1:5)
FITC CD16 BD Biosciences 3G8 cat. 555406 lot. 49228 (Dilution 1:5)
BB515 CXCR5 (CD185) BD Biosciences RF8B2 cat. 564624 lot. 9212785 (Dilution 1:20)
APC CD19 BD Biosciences HIB19 cat. 555415 lot. 9198515 (Dilution 1:5)
Alexa 647 CD127 BD Biosciences HIL-7R-M21 cat. 558598 lot. 8319700 (Dilution 1:5)
Alexa 647 CX3CR1 BD Biosciences 2A9-1 cat. 565895 lot. 8066688 (Dilution 1:20)
APC CD69 BD Biosciences FN50 cat. 560711 lot. 10330 (Dilution 1:20)

PE CD38 BD Biosciences HIT2 cat. 555460 lot. 21035 (Dilution 1:5)
PE ICOS (CD278) BD Biosciences DX29 cat. 557802 lot. 9352997 (Dilution 1:5)
PE CD141 BD Biosciences 1A4 cat. 559781 lot. 9162644 (Dilution 1:5)
PE CD66b BD Biosciences G10F5 cat. 561650 lot. 163100 (Dilution 1:20)
PE CD137 (4-1BB) BD Biosciences 4B4-1 cat. 555956 lot. 247889 (Dilution 1:5)
PE-Cy7 HLA-DR BD Biosciences G46-6 cat. 560651 lot. 9124 (Dilution 1:20)
PE-Cy7 CD19 BD Biosciences SJ25C1 cat. 557835 lot. 48190 (Dilution 1:20)
PE-Cy7 CD25 BD Biosciences 2A3 cat. 335789 lot. 9343937 (Dilution 1:20)
PE-Cy7 CD45RA BD Biosciences L48 cat. 337167 lot. 121372 (Dilution 1:20)
BV421 IgM BD Biosciences G20-127 cat. 562618 lot. 9002718 (Dilution 1:20)
BV421 PD-1 (CD279) BD Biosciences MIH4 cat. 564323 lot. 9346016 (Dilution 1:20)
BV421 CD303 BD Biosciences V24-785 cat. 566427 lot. 9296610 (Dilution 1:20)
BV421 CD56 BD Biosciences NCAM 16 cat. 562751 lot. 9350422 (Dilution 1:20)
BV421 CCR7 BD Biosciences 2-L1-A cat. 566743 lot. 56446 (Dilution 1:20)
BV421 Ki-67 BD Biosciences B56 cat. 562899 lot. 16592 (Dilution 1:20)
Alexa 647 Perforin BD Biosciences δG9 cat. 563576 lot. 9185574 (Dilution 1:20)
BV421 Granzyme B BD Biosciences GB11 cat. 563389 lot. 9344067 (Dilution 1:20)
PE-Cy7 CD3 BD Biosciences SK7 cat. 557851 lot. 79794 (Dilution 1:20)
APC-H7 CD8 BD Biosciences SK1 cat. 560179 lot. 41153 (Dilution 1:20)
FITC IFNγ BD Biosciences 4S.B3 cat. 554551 lot. 9282638 (Dilution 1:100)
APC TNF BD Biosciences MAb11 cat. 554514 lot. 76751 (1:30)
PE IL-17 BD Biosciences SCPL1362 cat. 560436 lot. 62225 (Dilution 1:5)
BD Horizon™ Fixable Viability Stain 510 BD Biosciences cat. 564406 lot. 9176704 (1:1000)
IgG HRP cat.IC-1H01 lot. 20075 (1:10000)
IgA HRP cat. A18781 lot. 61-153-050620 (1:10000)

Validation

Validation of antibodies is available at the manufacturer's website. Also all antibodies used in this study were previously tested before use to validate our eficiency.

Human research participants

Policy information about studies involving human research participants

Population characteristics

A prospective cohort study was carried out at Hospital Moinhos de Vento and at Hospital Restinga e Extremo Sul, both in Porto Alegre, southern Brazil. A cohort of 92 patients (25 children; 34 adults with mild disease - AMD; and 33 adults with severe disease – ASD). Median age was 9 for children, 37.8 for AMD and 60.8 for ASD. Mainly of female sex and Caucasian. All patients' characteristics are presented in table 1

Recruitment

A convenience sample of adults and children older than 2 months were enrolled from June to December 2020 at either the outpatient clinics (OPC), emergency rooms (ER), or hospitalized.Subjects were screened if presenting cough and/or axillary temperature ≥37.8ºC and/or sore throat. Both blood samples and respiratory samples collected through nasopharyngeal swabs were obtained at enrollment. Only patients with the clinical diagnosis of COVID-19 and SARS-CoV-2 infection confirmed by RT-PCR were included in the study. Clinical and demographic data were collected at inclusion, following a standardized protocol. Disease severity was classified according to the World Health Organization classification after completing the follow-up questionnaire. In this study there was no self-selection bias in the recruitment of the participants

Ethics oversight

This study was approved by the Institutional Review Board (IRB 30749720.4.1001.5330) at Hospital Moinhos de Vento and by the Ethics Committee at Universidade Federal de Ciências da Saúde (CAAE 30749720.4.3001.5345). Legal consent was obtained from all participants or their legal guardians. The study was conducted according to good laboratory practices and following the Declaration of Helsinki.

Note that full information on the approval of the study protocol must also be provided in the manuscript.

Flow Cytometry

Plots

Confirm that:

- [x] The axis labels state the marker and fluorochrome used (e.g. CD4-FITC).
- [x] The axis scales are clearly visible. Include numbers along axes only for bottom left plot of group (a 'group' is an analysis of identical markers).
- [x] All plots are contour plots with outliers or pseudocolor plots.
- [x] A numerical value for number of cells or percentage (with statistics) is provided.

Methodology

Sample preparation

Both blood samples and respiratory samples collected through nasopharyngeal swabs were obtained at enrollment. We

collected plasma and peripheral blood mononuclear cells (PBMCs) from adult and pediatric COVID-19 patients. Blood was collected in EDTA tubes (Firstlab, PR, Brazil) and stored at room temperature before processing for PBMC isolation and plasma collection. Plasma was separated by centrifugation and cryopreserved. PBMCs were next isolated by density-gradient centrifugation using Ficoll–Paque™ PLUS (GE Healthcare®), and either studied directly or resuspended in FBS 5% DMSO and stored in liquid nitrogen until use. For flow cytometry, cells were thawed by diluting them in 5mL pre-warmed complete RPMI1640 medium (Sigma-Aldrich - R8758) containing 5% FBS and spun at 1500 rpm for 5 minutes. Supernatants were carefully removed, and cells were resuspended in PBS. After, were stained with the BD Horizon™ Fixable Viability Stain 510 together with antibodies for surface markers.

Instrument	BD FACS Canto II
Software	Flow cytometry data was collected using a FACS-Canto II Becton Dickinson; the acquisition software was FACSDiva v.9.0. Data analysis was performed using FlowJo V10.7.1 (Becton Dickinson).
Cell population abundance	No sorting was performed in this study
Gating strategy	All gating strategy with FSC/SSC starting cell population gates and boundaries between "positive" and "negative" staining used in this study are presented in Supplementary Figura 1, in Supplementary Info file.

☒ Tick this box to confirm that a figure exemplifying the gating strategy is provided in the Supplementary Information.
